# Supplementary material for: Causal Relationship between Aging and Anorexia Nervosa: A White-Matter-Microstructure-Mediated Mendelian Randomization Analysis
Source: Biomedicines. 2024 Aug 16;12(8):1874. doi: 10.3390/biomedicines12081874 (PMC11351342; doi:10.3390/biomedicines12081874)
Supplement: Supplementary file 1 [file biomedicines-12-01874-s001.zip › Supplementary Figure/Figure S4/Singe SNP plot of MR analysis for TL to ubm-b-1526.pdf]

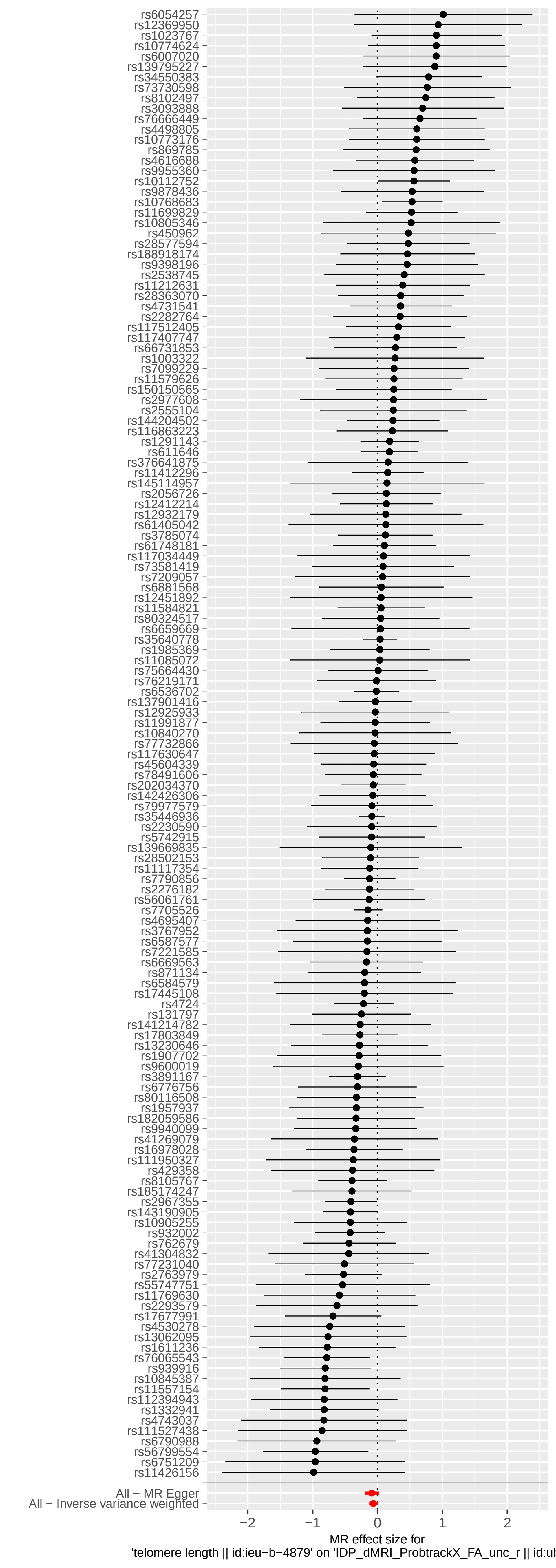
rs6054257
rs12369950
rs1023767
rs10774624
rs6007020
rs139795227
rs34550383
rs73730598
rs8102497
rs3093888
rs76666449
rs4498805
rs10773176
rs869785
rs4616688
rs9955360
rs10112752
rs9878436
rs10768683
rs11699829
rs10805346
rs450962
rs28577594
rs188918174
rs9398196
rs2538745
rs11212631
rs28363070
rs4731541
rs2282764
rs117512405
rs117407747
rs66731853
rs1003322
rs7099229
rs11579626
rs150150565
rs2977608
rs2555104
rs144204502
rs116863223
rs1291143
rs611646
rs376641875
rs11412296
rs145114957
rs2056726
rs12412214
rs12932179
rs61405042
rs3785074
rs61748181
rs117034449
rs73581419
rs7209057
rs6881568
rs12451892
rs11584821
rs80324517
rs6659669
rs35640778
rs1985369
rs11085072
rs75664430
rs76219171
rs6536702
rs137901416
rs12925933
rs11991877
rs10840270
rs77732866
rs117630647
rs45604339
rs78491606
rs202034370
rs142426306
rs79977579
rs35446936
rs2230590
rs5742915
rs139669835
rs28502153
rs11117354
rs7790856
rs2276182
rs56061761
rs7705526
rs4695407
rs3767952
rs6587577
rs7221585
rs6669563
rs871134
rs6584579
rs17445108
rs4724
rs131797
rs141214782
rs17803849
rs13230646
rs1907702
rs9600019
rs3891167
rs6776756
rs80116508
rs1957937
rs182059586
rs9940099
rs41269079
rs16978028
rs111950327
rs429358
rs8105767
rs185174247
rs2967355
rs143190905
rs10905255
rs932002
rs762679
rs41304832
rs77231040
rs2763979
rs55747751
rs11769630
rs2293579
rs17677991
rs4530278
rs13062095
rs1611236
rs76065543
rs939916
rs10845387
rs11557154
rs112394943
rs1332941
rs4743037
rs111527438
rs6790988
rs56799554
rs6751209
rs11426156
All − MR Egger
All − Inverse variance weighted
−2
−1
0
1
2
MR effect size for
'telomere length || id:ieu−b−4879' on 'IDP_dMRI_ProbtrackX_FA_unc_r || id:ub
